# Experimental Validation of Realistic Measurement Setup for Quantitative UWB-Guided Hyperthermia Temperature Monitoring

**DOI:** 10.3390/s24185902

**Published:** 2024-09-11

**Authors:** Alexandra Prokhorova, Marko Helbig

**Affiliations:** Biosignal Processing Group, Technische Universität Ilmenau, 98693 Ilmenau, Germany

**Keywords:** ultra-wideband microwave imaging, experimental validation, MIMO system, phantom measurements, temperature monitoring, hyperthermia, M-sequence radar technology

## Abstract

Hyperthermia induces slight temperature increase of 4–8 °C inside the tumor, making it more responsive to radiation and drugs, thereby improving the outcome of the oncological treatment. To verify the level of heat in the tumor and to avoid damage of the healthy tissue, methods for non-invasive temperature monitoring are needed. Temperature estimation by means of microwave imaging is of great interest among the scientific community. In this paper, we present the results of experiments based on ultra-wideband (UWB) M-sequence technology. Our temperature estimation approach uses temperature dependency of tissue dielectric properties and relation of UWB images to the reflection coefficient on the boundary between tissue types. The realistic measurement setup for neck cancer hyperthermia considers three antenna arrangements. Data are processed with Delay and Sum beamforming and Truncated Singular Value Decomposition. Two types of experiments are presented in this paper. In the first experiment, relative permittivity of subsequently replaced tumor mimicking material is estimated, and in the second experiment, real temperature change in the tumor imitate is monitored. The results showed that the presented approach allows for qualitative as well as quantitative permittivity and temperature estimation. The frequency range for temperature estimation, preferable antenna configurations, and limitations of the method are indicated.

## 1. Introduction

Even with ongoing advancements in medical science and the development of new technologies, aiming to enhance clinical diagnosis accuracy and treatment efficacy, malignant tumors are still among the leading causes of death [1]. Cancer treatment has been a crucial part of medical research for a long time as it is nowadays. There are so called “golden standard” approaches, such as surgery—the direct removal of the cancerous tissue via invasive operation, radiotherapy (RT)—the use of high doses of radiation to destroy cancer cells and shrink tumors, and chemotherapy (ChT)—the use of cytotoxic drugs to kill fast growing cancer cells. They can be used independently as well as in combination with each other or with some modifications. Combined treatments target cancer from multiple angles and therefore are more successful, offering higher survival rate together with better life quality. Supplementary cancer treatment approaches include but are not limited to hormonal therapy, immunotherapy, targeted therapy, gene therapy, and stem cell transplantation [2].

Another supplementary approach to oncological treatment is the application of hyperthermia (HT) therapy in combination with RT and/or ChT. Temperature increase just by 4–8 °C (hyperthermia temperature range) in the tumor region has shown a number of benefits on patients’ cure [3,4,5,6,7,8]. The heat application has direct biological effects such as damage of cancerous cells and shrinkage of the tumor size along with increased radiosensitivity, higher response to antigens and drugs in combination with ChT [9]. Moreover, addition of HT to the treatment plan can result in reduction in the amount of required RT and ChT sessions or in reduced dosage of radiation and chemo drugs, which certainly decreases overall toxicity of the oncological treatment.

One of the biggest obstacles of the expanded application of hyperthermia in clinical practice is temperature monitoring. In order to deliver the required amount of heat inside the tumor while avoiding overheating and thereafter damage of the surrounding healthy tissue, temperature in the treated region must be accurately controlled.

Currently, the most common ways of temperature measurement in medical applications are invasive temperature sensors and minimally-invasive endoluminal thermometry. In order to measure temperature during hyperthermia with invasive probes, they have to be inserted in plastic catheters or biopsy needles under imaging guidance to ensure proper positioning, which is time consuming and provides only limited point-like temperature data. Endoluminal thermometry, unfortunately, can be considered only for the limited types of cancer, since temperature probe can be inserted only in the hollow cavities such as rectum, cervix or urethra. Main medical concerns reported by the clinicians include risk of internal bleeding and infection [10]. Considering that hyperthermia therapy will be applied in weekly sessions for several months, non-invasive temperature monitoring would be advisable to improve the patient’s comfort, decrease the possibility of medical risks, as well as to reduce the treatment complexity.

Among the developing non-invasive approaches for temperature monitoring, the following can be distinguished: HT treatment planning and simulation-guided HT [11], magnetic resonance imaging (MRI) [12], computed tomography (CT) [13], ultrasound technology [14], electrical impedance tomography [15], passive [16,17] and active microwave imaging (MWI).

Microwave imaging is well suitable for medical applications since it avoids health risks due to non-ionizing radiation and is profitable in contrast with other imaging modalities, e.g., MRI and CT. Active MWI can be divided into microwave tomography (MWT) and ultra-wideband (UWB) imaging. Its working principle is based on the electromagnetic radiation of the body part to be treated and analysis of the transmitted and reflected signals.

During MWT, the transmitting antennas transmit single- or multi-frequency electromagnetic signals, which are scattered inside the medium under test and recorded by the receiving antennas. These signals are used as an input for specialized algorithms to create images of the distribution of dielectric properties representing the various tissues. In other words, microwave tomography directly provides estimated information on permittivity and conductivity of the tissues under test and is often referred to as the quantitative microwave imaging approach. MWT is based on the solution of the electromagnetic scattering problem and solves the inverse problem by iterative minimization of the difference between measured and simulated field values at the selected frequencies. Miscellaneous research groups investigated MWT for medical applications including temperature monitoring [18,19,20,21,22,23,24].

In contrast to MWT, in UWB radar-based MWI strong scatterers are detected and localized by means of coherent summation of the reflected signal energy. In relation to non-invasive temperature monitoring, UWB imaging intends to reconstruct tissue reflectivity, which varies due to the temperature-induced changes in the tissue dielectric properties. In comparison to MWT systems, UWB imaging systems have several notable advantages, such as lower price and shorter recording time compared to vector network analyzers used for MWT. Most importantly, UWB approach has lower computational effort and is not time consuming since it does not require the iterative solution of inverse scattering problem and is suitable for real-time three-dimensional (3D) applications. When required by the imaging type, UWB data can be preprocessed and analyzed in the frequency domain, as well. On the other hand, the method originally provides only qualitative images of the reflected signals from the region of interest, without estimation of the tissue dielectric properties.

The non-invasive temperature monitoring method presented in this paper is based on UWB M-sequence technology developed in Technische Universität Ilmenau [25]. Our methodology was previously published in [26], where it was for the first time experimentally tested on a preliminary measurement setup for breast cancer detection [27] and images were reconstructed via standard beamforming imaging algorithm (IA). It was further followed up in our work [28], where the phantom, specifically tumor tissue mimicking materials, was prepared to represent HT-induced changes in the dielectric properties more accurately, and two types of IA were applied to the measured data, including a comparison of their performance. Later, we investigated the requirements and limitations for a hybrid system for MWI-guided HT and designed a measurement setup of an anatomically realistic shape representing the human neck with a reasonably reduced amount of measurement channels [29]. This setup was additionally tested in [30], providing first exemplary imaging results.

In this paper, we present a comprehensive experimental validation of this methodology on the measurement setup mentioned above with optimized and practically applicable arrangements of antennas for a hybrid MWI system for non-invasive temperature monitoring during hyperthermia. Moreover, we not only measure and estimate the permittivity of the tumor imitates but perform radar measurements during real temperature change inside the tumor and analyze these data quantitatively. Additionally, new tumor mimicking materials are introduced, which, besides well representing the dielectric properties of the tumor tissue in the HT temperature range, also show high stability over a long period of time which is required for repeatable measurements.

The remainder of the paper is organized as follows. Section 2 describes the working principle and methodology of UWB MWI, especially the IA and temperature estimation approach applied in this work, as well as the description of the experimental setup, relevant phantom materials, and measurement protocols. Exemplary imaging and estimation results for two types of experiments as well as analysis and review of the whole measured dataset are presented in Section 3. We conclude and discuss our findings and compare them with our previously published results in Section 4.

## 2. Materials and Methods

### 2.1. Frequency and Temperature Dependent Dielectric Properties

The dielectric properties of biological tissues are the physical basis for microwave imaging, since they determine the tissue interaction with electromagnetic fields. They are described by the complex relative permittivity $\underline{\varepsilon }$ and the effective conductivity σ as:

(1) $$\begin{array}{c} \underline{\varepsilon }(\omega ,\vartheta )=\varepsilon^{\prime }(\omega ,\vartheta )-i\varepsilon^{\prime \prime }(\omega ,\vartheta )\\ \sigma (\omega ,\vartheta )=\omega \varepsilon_{0}\varepsilon^{\prime \prime }(\omega ,\vartheta ) \end{array},$$

where the real part $\varepsilon^{\prime }$ represents the relative permittivity, the imaginary part $\varepsilon^{\prime \prime }$ the relative dielectric loss, i the imaginary unit, $\varepsilon_{0}$ the permittivity of free space, ω the angular frequency, and ϑ the temperature.

The range of dielectric parameters depends on the water content of the tissue. In principle, all human tissues can be divided into low water content (LWC) and high water content (HWC) ones. HWC tissues include, for example, blood, gland, muscle or liver, while LWC tissues are represented mostly by fat and bone. The study [31] well demonstrates that the amount of water content present in biological tissues has a direct effect on the values of their dielectric properties. The higher the water content in the tissue—the higher its permittivity. For example, due to the increased blood flow, most tumors are HWC tissues with quite high relative permittivity. Based on the available data, the permittivity of tumor tissues in the frequency range of interest belongs to the interval 55–70 [32,33,34,35,36].

The magnitude of changes in dielectric parameter during HT depends on the tissues’ water content, as well. HWC tissues exhibit larger changes in the relative permittivity as a function of temperature in comparison to LWC tissues. A monotonic decreasing trend can be observed with increasing temperature. The quantitative temperature dependency of blood and liver is close to the tumor [37,38,39,40]. The permittivity decrease is approximately 0.20 per degree Celsius for blood and around 0.10 for liver. On the other hand, fat tissue, whose parameters can be used to mimic the healthy tissue surrounding the tumor, is practically temperature independent due to its LWC. The decrease in relative permittivity is not more than 0.02 per degree Celsius between 1 GHz and 7 GHz [41]. Based on the tissue types of the body region to be monitored, these values can be applied for the conversion of the estimated permittivity changes to temperature increase.

### 2.2. Methodology

**Figure 1:**
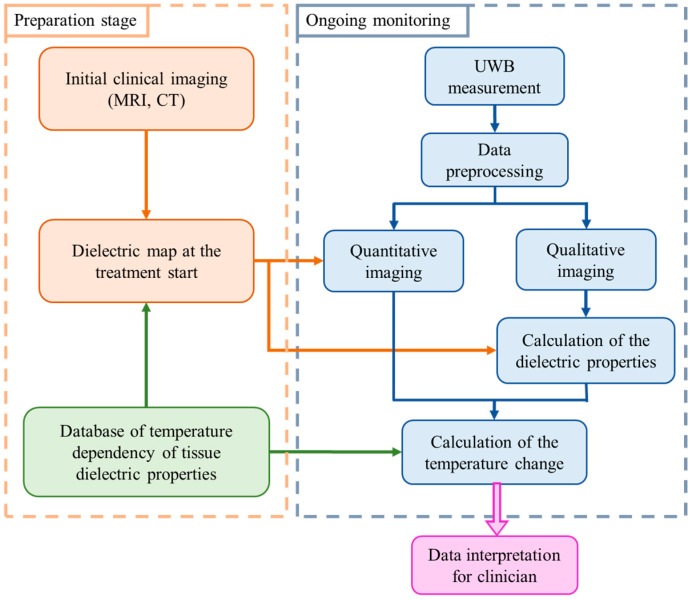
Flowchart of the tissue temperature monitoring methodology [26].

In general, our methodology for non-invasive temperature monitoring during hyperthermia is based on continuous estimation of the changing tumor tissue dielectric properties by means of microwave sensing [26]. The flowchart illustrating this approach is shown in Figure 1.

The preparation stage indicates the steps to be completed prior to the hyperthermia session. Relying on the clinical image obtained by another modality, an individual dielectric map is created for each patient. All tissues present in the region to be treated are assigned values of relative permittivity and effective conductivity using the database of tissue specific dielectric properties and their temperature dependency. These dielectric tissue maps of the region of interest are used as input (initial guess) for microwave imaging.

Ongoing monitoring of temperature via UWB technology is performed in real-time alongside the thermal therapy. Changes in the dielectric properties are directly reconstructed via imaging algorithms or by applying an estimation approach exploiting changes in UWB images. Lastly, the change in the dielectric properties in the cancerous region is converted to the change in temperature in degree Celsius.

### 2.3. Signal Processing

The received UWB measurement signal of channel *n* can be written as:

(2) $$s_{n}(t)=s_{c,n}(t)+s_{h,n}(t),$$

where *t* is the signal propagation time, $s_{c,n}(t)$ is the static clutter including channel specific antenna crosstalk as well as the static signal components caused by reflections inside the radar measurement system, and $s_{h,n}\left(t\right)$ is the ongoing and patient specific signal due to heating.

**Figure 2:**
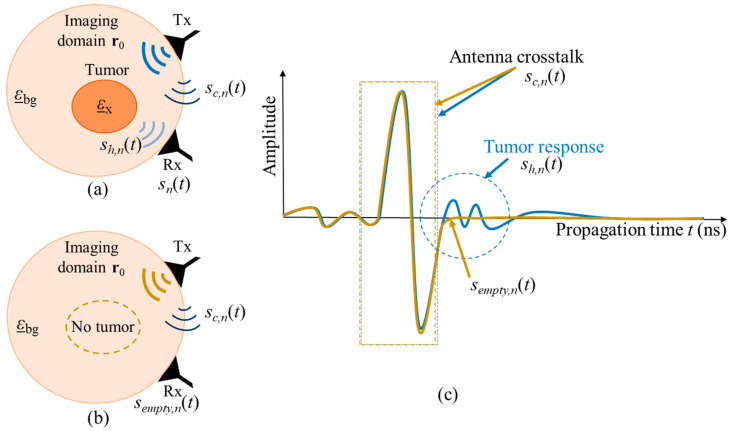
Schematic of two measurement scenarios (**a**) with tumor and (**b**) without tumor. The corresponding UWB signals from both measurements are presented in (**c**).

The amplitude of the crosstalk between antennas is significantly larger than the scattering from the inside of the neck, thus, it has to be eliminated. One simple option is the creation of the same scenario under test but without the target, in our case—tumor. In literature, this scenario is often referred to as empty measurement. After recording signals from both scenarios, the empty measurement signal will be subtracted from the signal of the scenario with tumor, and therefore the crosstalk, which is stable throughout both measurements, is eliminated. Figure 2 illustrates both measurement scenarios and the corresponding received signals, where ${\underline{\varepsilon }}_{x}$ is the complex permittivity of the tumor, ${\underline{\varepsilon }}_{bg}$ is the complex permittivity of the surrounding background medium, and $r_{0}$ is the focal point inside the imaging domain. Transmitting and receiving microwave antennas are represented by Tx and Rx, respectively. It is important to note that, this way of crosstalk removal is not possible in real clinical scenarios, but it can be used for investigations under laboratory conditions, e.g., for proof of concept or methodology validation.

**Figure 3:**
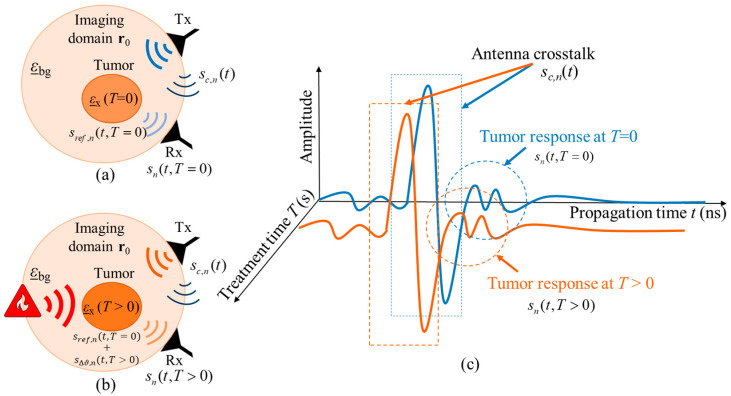
Schematic of the measurement scenario of temperature estimation during hyperthermia by means of UWB sensing (**a**) at the start of treatment and (**b**) during the treatment. The corresponding UWB signals from both treatment stages are presented in (**c**).

In the case of real hyperthermia monitoring, the received UWB measurement signal of channel *n* is also dependent on the HT treatment time *T*, so that $s_{n}(t,T)=s_{c,n}(t)+s_{h,n}(t,T).$ After crosstalk removal, the monitoring relevant signal part remains as:

(3) $$s_{h,n}\left(t,\;T\right)=s_{ref,n}\left(t,T=0\right)+s_{\Delta \vartheta ,n}\left(t,\;T\right),$$

where $s_{ref,n}\left(t,T=0\right)$ is the reference signal representing the tissue region to be treated at the beginning of hyperthermia session and $s_{\Delta \vartheta ,n}\left(t,T\right)$ is the varying signal representing the change in the dielectric properties with ongoing treatment time *T* due to the changing tissue temperature. Figure 3 illustrates the hyperthermia measurement scenario, including the related signals.

### 2.4. Microwave Imaging Algorithms

Two algorithms which are used for imaging in this paper are Delay and Sum (DAS) and Truncated Singular Value Decomposition (TSVD) [21]. More details on the application of these imaging algorithms for our approach can be found in [28].

DAS algorithm is based on the principle of coherent addition of backscattered radar signals, which are collected while illuminating the target with electromagnetic waves. The main advantages of this algorithm are its simplicity, robustness, and short computational time. The DAS beamformer equation can be written as:

(4) $$I_{DAS}(r_{0},T)=\left|\sum_{n=1}^{N}s_{h,n}\left(t_{0}+\tau_{n}(r_{0}),T\right)\right|,$$

where *I* represents the image intensity, *N* is the number of channels (pairs of Tx and Rx antennas), $t_{0}$ is the time when the electromagnetic wave takes off the transmitter antenna, and $\tau_{n}$ is the channel dependent time of flight—distance from the transmitting antenna to the focal point $r_{0}$ inside the imaging domain and back to the receiving antenna. The signals received at each channel are time-aligned for each focal point and coherently summed. The energy of the summed signal is assigned to the reflection intensity of the focal point and this process is repeated for all focal points within the region of interest.

In contrast to our previous studies [26,28], where the antennas could be rotated around the phantom, the imaging in this paper is based on HT realistic measurements with stationary antennas, which significantly reduces the number of received signals to be summed. Nevertheless, in order to stabilize the subsequent permittivity estimation, the envelope of $\;s_{h,n}\left(t,T\right)$ is used in this article:

(5) $$I_{DAS}^{env}(r_{0},T)=\sum_{n=1}^{N}\left|s_{h,n}\left(t_{0}+\tau_{n}(r_{0}),T\right)+i\cdot H\left(s_{h,n}\left(t_{0}+\tau_{n}(r_{0}),T\right)\right)\right|,$$

where *H* is the Hilbert transform. In comparison to Equation (4), the imaging loses resolution but gains robustness due to the avoided mutual extinction of non-perfectly aligned channels.

TSVD is commonly used in MWT, however, it can be easily adapted for UWB radar-based imaging. The linear inverse problem is represented:

(6) $$A(r_{0})b(r_{0},T)=S_{h}(T),$$

where *A* is the scattering operator, mapping the unknown contrast function *b* at different treatment time *T* into the frequency domain data $S_{h}\left(T\right)$, obtained as the Fourier Transform of $s_{h}(t,T)$. For each channel, the scattering operator *A* is represented by the discretized version of a linear and compact integral operator. The implementation of the TSVD algorithm requires the prior knowledge of the incident field in the reference scenario (start of treatment at *T* = 0). The more accurate the model of the reference scenario, the more reliable images can be obtained via this reconstruction technique.

In order to retrieve the solution from the data, the scattering operator *A* must be inverted. To this end, its representation in terms of singular value decomposition can be exploited as $A^{+}=VD^{+}U^{*},$ where symbol ^+^ denotes the inverse of the matrix, *V* represents right singular vectors, *U* denotes left singular vectors, and *D* is a diagonal matrix, where the main diagonal is non-zero, with decreasing singular values. Since the inverse scattering problem is ill-posed, a regularized solution is necessary to retrieve reliable images. Hence, a truncated singular value decomposition scheme is adapted, where only a limited number of singular functions is considered in the reconstruction formula:

(7) $$I_{TSVD}(r_{0},T)=\left|\sum_{n=1}^{N}V_{C,n}\cdot D_{C,n}^{+}\cdot U_{C,n}^{*}\cdot S_{h,n}(T)\right|,$$

where *C* represents the regularizing truncation parameter, which is chosen to meet a good tradeoff between accuracy and stability of the reconstruction. It is worth noting that the singular value decomposition of the scattering operator can be computed off-line at the preparation stage (Figure 1), which is time-efficient and qualifies this algorithm for real-time medical applications.

### 2.5. Tumor Temperature Estimation

The tumor temperature estimation algorithm is based on the temperature dependent changes in the reflection coefficient at the boundary between the tumor and surrounding tissue. The image values $I\left(r_{0},T\right)$ in the region of treatment are presumed to depend on two parts: the dielectric contrast between the tumor and surrounding tissue as well as on the accumulation of temperature independent influencing parameters (e.g., radar cross section of the tumor, signal path attenuation, impulse response of radar and antennas, etc.), which are not known exactly, and cannot be quantified separately. All these factors are included in parameter $\underline{F}\left(r_{0}\right)$, which remains constant during the hyperthermia treatment. Specular reflection is assumed at the boundary between the background medium and the tumor, so that the effect of dielectric contrast can be approximated by means of the complex reflection coefficient $\underline{\Gamma }$. Thus, image intensity is related to the reflection coefficient as: $I(r_{0},T)=\underline{F}(r_{0})\underline{\Gamma }(r_{0},\omega ,T)$. Based on the initial clinical imaging and the database of tissue specific dielectric properties, the permittivity of tumor ${\underline{\varepsilon }}_{x}$ and permittivity of healthy tissue ${\underline{\varepsilon }}_{bg}$ at the beginning of treatment are assumed to be known. During the treatment, we relate the ongoing images to the reference one (*T* = 0), along with the corresponding reflection coefficients, and accordingly eliminate the effect of $\underline{F}\left(r_{0}\right)$:

(8) $$\frac{I(r_{0},T)}{I_{ref}(r_{0},T=0)}=\frac{\underline{\Gamma }(r_{0},\omega ,T)}{{\underline{\Gamma }}_{ref}(r_{0},\omega ,T=0)}.$$

Lastly, the changing permittivity of the tumor ${\underline{\hat{\varepsilon }}}_{x}(r_{0},\omega ,T)$ can be quantitatively estimated as:

(9) $${\underline{\hat{\varepsilon }}}_{x}(r_{0},\omega ,T)={\underline{\varepsilon }}_{bg}(\omega ){\left(\frac{I_{ref}(r_{0},T=0)-{\underline{\Gamma }}_{ref}(r_{0},\omega ,T=0)I(r_{0},T)}{I_{ref}(r_{0},T=0)+{\underline{\Gamma }}_{ref}(r_{0},\omega ,T=0)I(r_{0},T)}\right)}^{2}.$$

It has to be emphasized that this approach estimates the permittivity and respectively the temperature only in the tumor region. This is because outside this area ${\underline{\Gamma }}_{ref}\left(r_{0,}\omega \right)=0$, so that Equation (9) yields ${\underline{\hat{\varepsilon }}}_{x}(r_{0},\omega ,T)={\underline{\varepsilon }}_{bg}(\omega )$ independently from $I\left(r_{0},T\right)$.

### 2.6. Measurement Setup and Measurements

#### 2.6.1. Ultra-Wideband Radar

Our radar hardware is based on the UWB M-sequence technology [25] applying pseudo-noise codes as a stimulation signal of sufficiently large bandwidth. The main benefits of M-sequence technology are long-term stability and low jitter. Since the impulse response is created by means of cross correlation between received signal and ideal M-sequence, the amplitude unit is arbitrary. In order to build complex multiple input multiple output (MIMO) systems, single sensor modules are cascaded. A baseband MIMO system (bandwidth 6.5 GHz) providing a maximum of 24 transmitters and 48 receivers [42] is used in this study.

#### 2.6.2. Antennas and Antenna Arrangements

The design of a hybrid microwave hyperthermia treatment system, which means inclusion of microwave-based heating as well as microwave-based temperature monitoring, states several obstacles. While interference issues resulting from similar operating frequencies of the system’s heating and imaging components can be largely avoided by achieving perpendicular polarization, quite small dimensions of the neck area present a challenge. Therefore, sensing antennas should have a small size and be applicable for quasi direct positioning. We use bow-tie antennas with dimensions of 10 by 5.6 mm, implemented on an FR4-printed circuit board material (0.8 mm thickness). Antennas are differentially fed by passive baluns. Three waveguide heating applicators of relatively large dimensions (10 by 5 cm) are necessary for efficient hyperthermia [43]. While the middle applicator, placed directly in front of the tumor, provides the main amount of heat, the other two applicators are set to focus heat transfer into the tumor. Since the space for the positioning of sensing and heating antennas is limited and sensing antennas cannot be placed above or below the heating applicators, 40% of the neck circumference is reserved for the hyperthermia applicators, 40–50% for sensing antennas, and the remaining space can be used for customization of the system to patient-specific neck diameters.

Three basic configurations defining the number of columns with sensing antennas between the three heating waveguide applicators, named and numerated as channel configurations (ChCnf 1 … 3) are specified. To enhance the quality of the image reconstruction, 32 antennas (12 Tx and 20 Rx) are used for these measurements instead of 20 antennas (8 Tx and 12 Rx) as reported in our previous publications [29,30].

**Figure 4:**
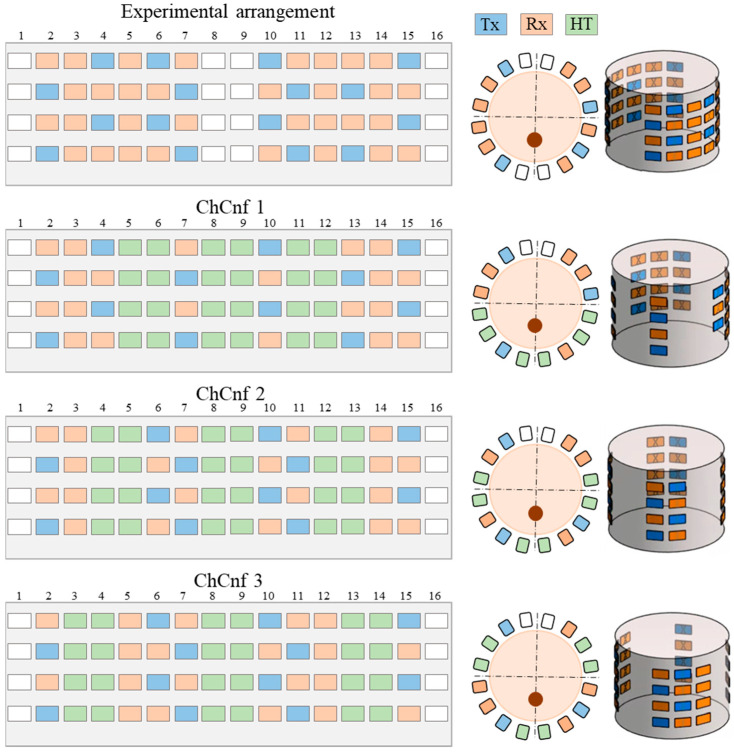
Unified experimental arrangement including 48 antennas (above) and three ways to arrange the heating antennas around the neck phantom resulting in three channel configurations, where the blue color represents transmitting antennas, orange slots indicate receiving antennas, the space for three hyperthermia applicators is represented in green color, and white color indicates empty slots. The figure shows the unfolded presentation (left) of the cylindrical 3D antenna arrangement (right) and xy-slice of the top row (center).

For practical reasons, we define a unified antenna arrangement scheme, consisting of 48 antennas (16 Tx and 32 Rx) and including all three ChCnf. This allows us to measure the data within one measurement and later split it into individual configurations in a preprocessing step. The exact antenna positions for the mentioned ChCnf are shown in Figure 4.

#### 2.6.3. Phantom, Tissue Mimicking Materials, and Measurement Setup

The corpus for the antenna array and the neck phantom is 3D printed from plastic filament material (polylactic acid). The corpus is designed to fit a cylindrical neck phantom while providing firm placement of sensing antennas in accordance with Figure 4, thus the outer shape is a 16-sided polygon. The neck phantom includes a cylinder filled with liquid material mimicking healthy tissue and a tube which imitates the tumor.

**Figure 5:**
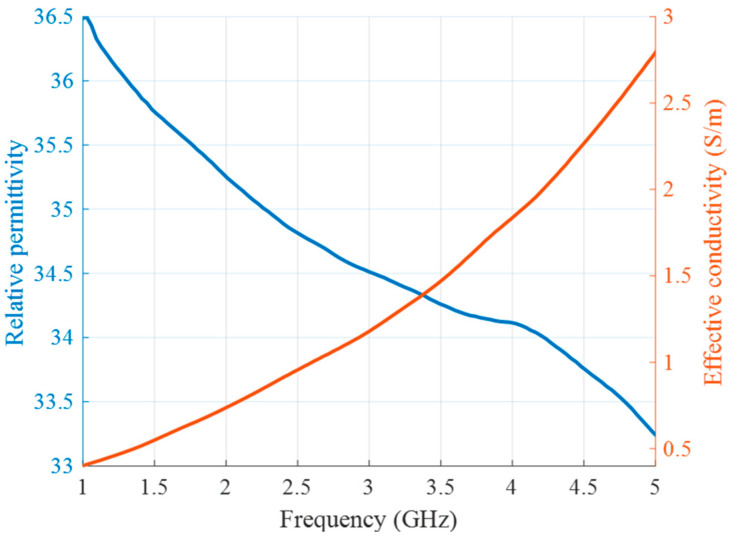
Relative permittivity (blue) and effective conductivity (red) of background medium (cream) as a function of frequency.

Since the human neck is mainly represented by muscle and fat tissues, the tumor surrounding material should have intermediate properties of these two tissue types [44,45]. Thus, for the background medium we use cream (32% fat), whose liquid physical state is appropriate for this phantom configuration. Its measured dielectric properties are presented in Figure 5.

To mimic HWC tissue like tumor, deionized distilled water and Triton X-100 (TX-100) are mixed in different proportions. As reported by Joachimowicz et al. in [46] and by Relva and Devesa in [47], TX-100 has a dielectric constant around 8–10 almost throughout the whole microwave frequency range, showing no strong frequency dispersion. Thus, it is chosen as a substance which decreases the relative permittivity of tumor mimicking materials.

**Figure 6:**
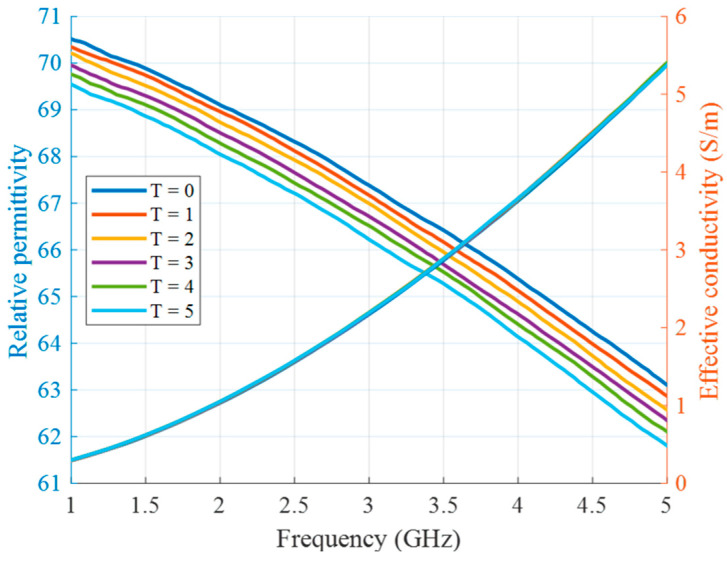
Relative permittivity and effective conductivity of tumor mimicking materials in the first experiment as a function of frequency.

Two types of experiments are presented in this article. The first type of experiment is based on manually changed tumor phantom material in order to mimic the increase in temperature during HT. The use of liquid tumor imitates in the test tube provides the opportunity for fast replacements and accurate manipulations without excessive interaction with the measurement setup. To this end, tumor mimicking materials are prepared based on the differentiating proportions of TX-100. Tumor at the beginning of the HT treatment (*T* = 0) is represented by 10 vol% of TX-100. Each next stage of heated tumor (*T* = 1 … 5) is mixed with an additional 0.2 vol% of TX-100, decreasing the permittivity of the tumor with ongoing HT. Figure 6 presents measurements of relative permittivity and effective conductivity of these tumor mixtures, performed based on coaxial probe S11 measurements using the UWB M-sequence network analyzer described in [36].

**Figure 7:**
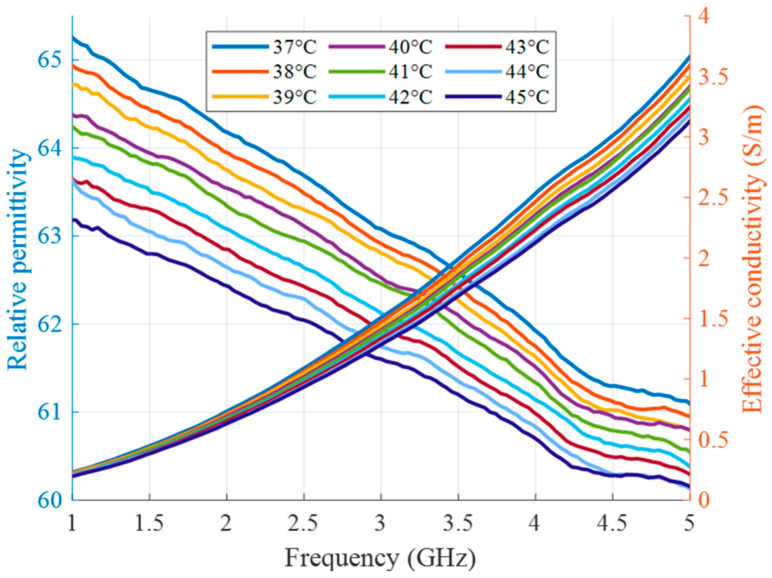
Relative permittivity and effective conductivity of tumor mimicking material in the HT temperature range as a function of frequency.

In the second type of experiment, the tumor imitating material remains the same (10 vol% of TX-100), but its temperature changes and will be eventually estimated. In this preliminary stage of measurement setup without microwave heating applicators, it is not the heating but the cooling of the material that is monitored in the experiment. The tumor mimicking material is heated up separately via hot plate and a water bath as described in our previous publications [36,41]. After insertion of the material into the tube, this process does not require any contact of the person conducting the experiment with the measurement setup and therefore excludes several errors related to this influence. Figure 7 shows the relative permittivity and effective conductivity of the tumor imitate depending on the frequency and temperature in the HT relevant range. These measurement data act as the “Database of temperature dependency of tissue dielectric properties”, as shown in Figure 1, in order to derive temperature values from the estimated permittivity values in this experiment.

We use fiber optic temperature sensors (FOTEMP Modular System MS-14TE, WEIDMANN Technologies Deutschland GmbH, Dresden, Germany) in parallel with radar measurements to monitor the temperature during the experiment in real-time and to validate our non-invasive temperature estimation. The sensors are completely non-conductive, immune to microwave radiation, have an accuracy of ±0.2 degree Celsius with a fast time response [48].

**Figure 8:**
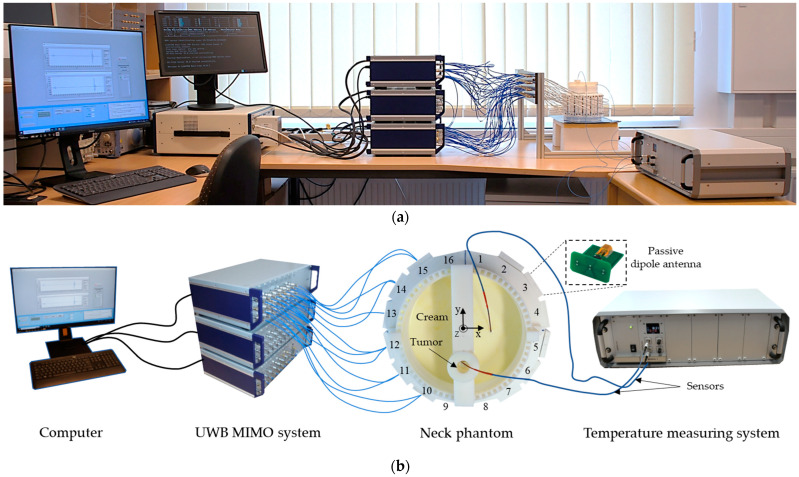
Measurement setup: photo (**a**), schematic (**b**).

A photo of the measurement setup is presented in Figure 8. It includes UWB MIMO M-sequence radar system (bandwidth 6.5 GHz, Ilmsens GmbH, Ilmenau, Germany), corpus with an array of 48 passive dipole antennas, neck phantom (Ø 13 cm) filled with background material, and plastic lab tube (Ø 1.5 cm) filled with tumor mimicking material. The exact tube position is x = 0 cm, y = −2.5 cm. The data acquisition of the MIMO system is realized by means of PXIe (PCI eXtensions for Instrumentation, National Instruments, State of Delaware, United States) system [42] and in-house developed software. For the second set of measurements, the temperature sensors are inserted in the tube and in the neck phantom.

## 3. Results

In this section, we present imaging and estimation results of two types of conducted experiments. In Section 3.1, the imitation of temperature-induced permittivity changes by the successive replacement of the tumor imitating material, and in Section 3.2, real temperature estimation of the tumor material during its cooling from 45 °C to 37 °C.

In both experiments, DAS- as well as TSVD-based images are calculated. The imaging domain of DAS is x = −6.5 … 6.5 cm, y = −6.5 … 6.5 cm, z = −10 … 0 cm with the resolution of 1 mm, resulting in 1.7 million voxels.

TSVD imaging is much more computationally intensive and sensitive to a set of interdependent parameters, e.g., frequency of the reconstruction, dimensions and resolution of the imaging domain in relation to the number of channels, model simplification, etc. Therefore, the imaging domain of TSVD-based imaging is set to focus on the tumor area with x = −2.5 … 2.5 cm, y = −5 … 0 cm, z = −5.5 … −3.5 cm with the resolution of 1 mm, resulting in approximately 55 thousand voxels. Reconstruction frequency is chosen empirically, based on our previous experiments, 1.5 GHz. The regularization parameter *C* is uniformly set to 8 satisfying that the threshold for singular values is usually set between −3 and −30 dB, which is considered optimal in MWI community for medical applications [49,50,51,52,53]. It is a compromise, trying to assure no over (excludes the informative part of the data) and under (contains errors caused by, e.g., model approximation) regularization. Furthermore, a manual check revealed that this value yields good overall estimation results as will be shown later (Figures 13 and 19).

Data preprocessing consists of crosstalk removal based on empty measurement, exclusion of plastic tube influence as described in [26], and splitting of the whole measured data record to configurations ChCnf 1 … 3. Then, data are processed by DAS and TSVD.

To achieve reliable and consecutive results, the measurement series are repeated over a period of eight weeks. The results are presented in a recurring manner. After presenting the images of one exemplary session and the subsequent permittivity or temperature estimation, respectively, the overall estimation is presented. This is performed separately based on DAS and TSVD.

### 3.1. Imaging and Estimation of the Manually Changed Permittivity

The measurement set consists of the insertion and replacement of six tumor mimicking liquids, described in Section 2.6, that imitate different heating stages from *T* = 0 (reference stage) to *T* = 5 (highest temperature) during the HT session.

**Figure 9:**
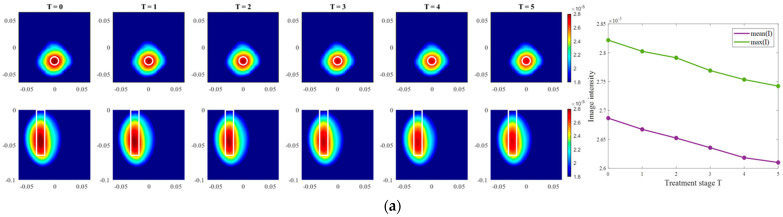

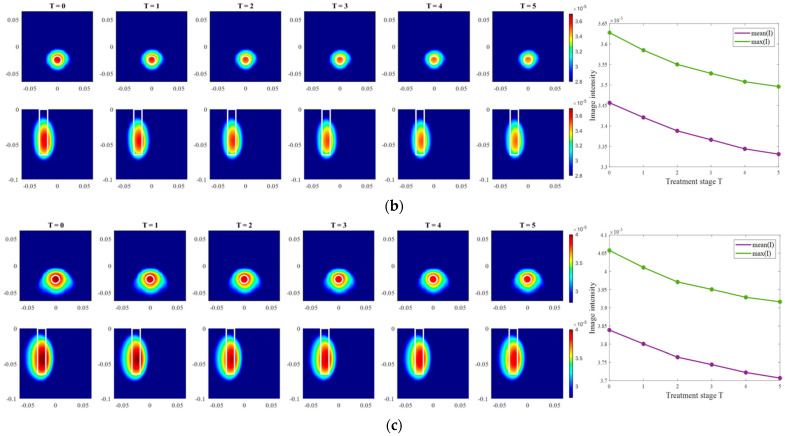
DAS-based images in xy-slice, z = −5 cm (upper row), in yz-slice, x = 0 cm (bottom row) and corresponding image metrics of the six mimicked HT stages for ChCnf 1 (**a**), ChCnf 2 (**b**), and ChCnf 3 (**c**). Color bars represent the intensity of the images in the arbitrary units. White lines indicate the shape and position of the tumor. The unit of the axis labeling is m.

Images of an exemplary measurement session, reconstructed via DAS (Equation (5)), are presented in Figure 9. They show the ability not only to localize the tumor, but also to detect the decreasing dielectric contrast due to decreasing permittivity of the tumor imitate. This can be deduced from the decreasing image intensity with increasing heating stage. In addition to the qualitative visual information of the images, the mean and maximum image values within the tumor region are plotted on the right side of Figure 9, respectively. As can be seen, the image values are decreasing with the ongoing treatment stage in a close to linear way for each of three channel configurations.

**Figure 10:**
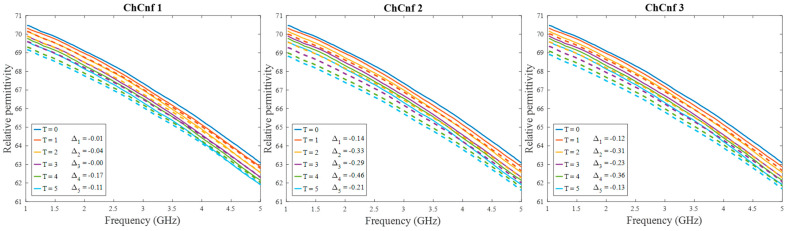
Via DAS estimated mean relative permittivity in the 3D tumor region (dashed lines) and measured values (solid lines) as a function of frequency. Differences between them at each stage at frequency 4.5 GHz are shown as Δ_T_.

After qualitative validation of the measurement, complex permittivity is estimated via Equation (9). In this paper, we concentrate on the temperature estimation based on the real part of complex permittivity. Therefore, Figure 10 presents measured and estimated values of relative permittivity over the frequency range 1–5 GHz. The estimated curves tend to be in good correspondence with the true curves.

**Figure 11:**
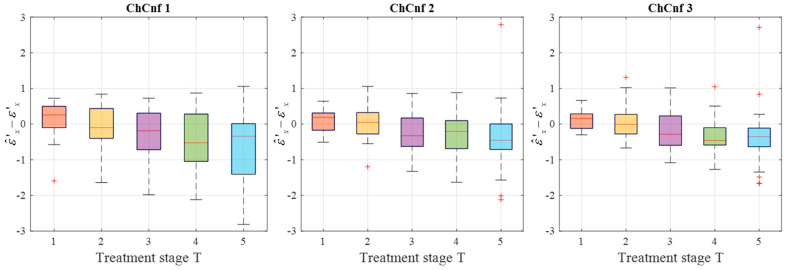
Differences between via DAS estimated and measured relative permittivity values at 4.5 GHz of all measurement sessions. Red lines indicate the median, red plus signs show the outliers.

To achieve a better understanding of the estimation reliability, 21 measurement series are recorded and the differences between the real (measured) permittivity and each estimated value are calculated. Figure 11 shows the boxplots (each box is an interquartile range including the middle 50% of all data, red lines indicate the median, and red plus signs show the outliers—values, which are 1.5 times bigger or smaller than the limits of the interquartile range) of all heating stages and channel configurations. It reveals that the permittivity estimation based on DAS already shows promising results. The interquartile ranges of the estimation mostly (except for *T* = 4 and *T* = 5 in ChCnf 1) extend within ±0.7 and in general increase with the ongoing treatment stage or monitoring time, respectively.

**Figure 12:**
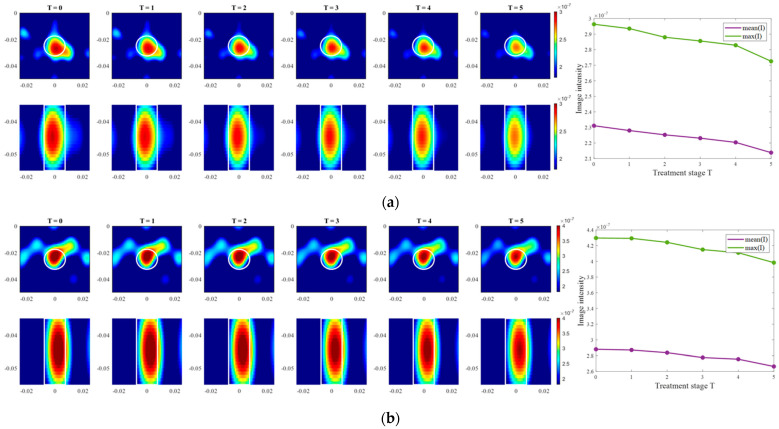

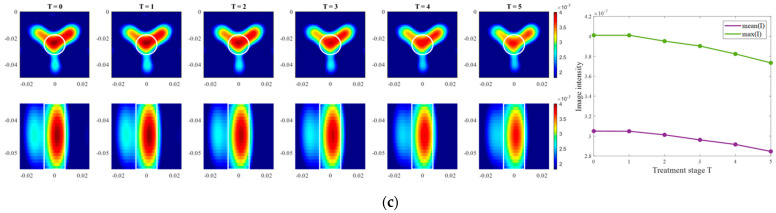
TSVD-based images in xy-slice, z = −4.5 cm (upper row), in xz-slice, y = −2.5 cm (bottom row) and corresponding image metrics of the six mimicked HT stages for ChCnf 1 (**a**), ChCnf 2 (**b**), and ChCnf 3 (**c**). Color bars represent the intensity of the images in the arbitrary units. White lines indicate the shape and position of the tumor. The unit of the axis labeling is m.

Moving on to the results acquired via inverse scattering, the images of the exemplary measurement session reconstructed via Equation (7) at the frequency 1.5 GHz are presented in Figure 12. The target is also localized correctly (even though there are visible elongated trails in reconstructions of ChCnf 3) and the intensity of the images in each row corresponds to the decreasing contrast between permittivity of the tumor imitate and the background material. The same inference is supported by the trend of mean and maximum intensities inside the tumor region with increasing treatment stage as shown in the right side of Figure 12.

**Figure 13:**
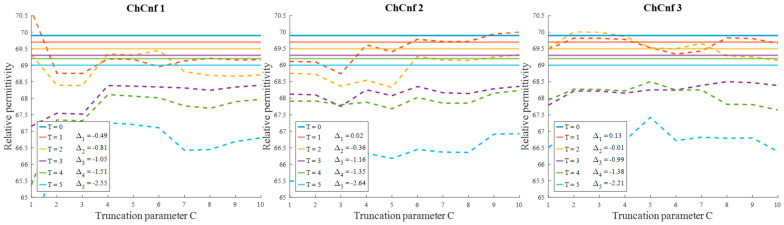
Via TSVD estimated mean relative permittivity at 1.5 GHz in the 3D tumor region (dashed lines) and measured values (solid lines) as a function of truncation parameter. Differences between them at each stage for *C* = 8 are shown as Δ_T_.

To evaluate the influence of the chosen truncation parameter *C* on the image reconstruction and consequently the accuracy of estimation, the dependency of the number of used singular values and the estimated permittivity is investigated and presented in Figure 13. Some dissimilarity can be observed between real and estimated permittivity values. Even though at the earlier stages of treatment the estimation, e.g., for ChCnf 2 and ChCnf 3 is almost correct, at the subsequent stages *T* = 3 … 5 the difference is noticeably increasing. In ChCnf 1 and ChCnf 3, the estimated curves at treatment stages *T* = 1 and *T* = 2 have a twist at *C* ≤ 7, which can lead to an error in interpretation. In ChCnf 2, regularization parameter *C* ≥ 9 causes a slight overestimation of permittivity at treatment stage *T* = 1. Thus, Figure 13 confirms that the optimal truncation parameter for estimation is equal to 8 singular values.

**Figure 14:**
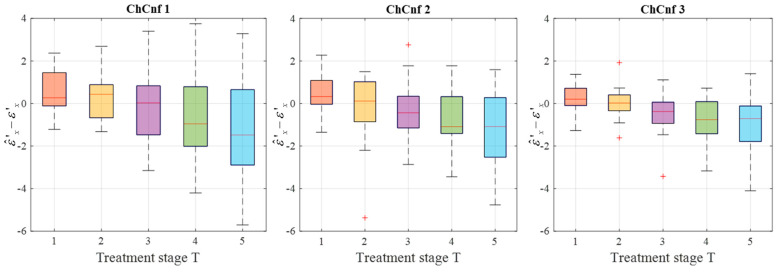
Differences between via TSVD estimated and measured relative permittivity values at 1.5 GHz of all measurement sessions. Red lines indicate the median, red plus signs show the outliers.

To see the dispersion of the TSVD-based permittivity estimation values over all measurement sessions, their boxplots are presented in Figure 14. Quantitative analysis of these estimations shows an interquartile spreading in the range −2 … 1 (except for *T* = 1 in ChCnf 1 and *T* = 5 in ChCnf 1 and ChCnf 2) as well as an overall increase in error with each subsequent treatment stage.

To summarize, the developed realistic measurement setup of a hybrid system for UWB-guided HT monitoring proves to be appropriate for non-invasive estimation of tumor dielectric properties, and accordingly temperature. The results from the first experiment show that very small variations of permittivity can be detected and qualitatively estimated by both IA. The image intensity in the 3D tumor region decreases with decreasing dielectric contrast between tumor imitate and background material. The quantitative estimations of the tumor permittivity via DAS have higher accuracy than the ones acquired via TSVD, which can be explained by the complexity and sensitivity of the inverse algorithm to parameters mentioned in Section 3. Additionally, in this type of experiment, signal distortions caused by direct interaction of the experimenter with the corpus of the measurement setup during the experiment cannot be ruled out completely.

### 3.2. Imaging and Estimation of Temperature

This section is dedicated to the radar measurements of the changes in the tumor dielectric properties caused directly by temperature. Cream, imitating surrounding healthy tissue, is preheated to 37 °C. Then, the tumor imitate heated to the temperature above 55 °C is poured into the target tube. Temperature sensors are positioned in advance in and outside the tube. Since we are interested specifically in the HT temperature range, as soon as the tumor temperature reaches 45 °C, UWB measurements will be started and continued in intervals of 1 °C until the average body temperature is reached. The cooling process of the tumor from 45 °C to 37 °C takes on average 7 to 10 min.

**Figure 15:**
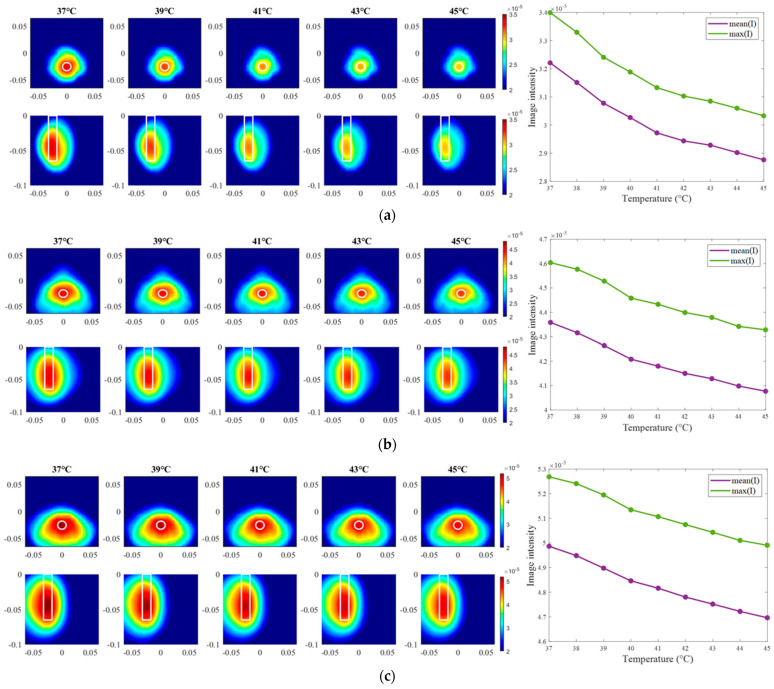
DAS-based images in xy-slice, z = −5 cm (upper row), in yz-slice, x = 0 cm (bottom row) and corresponding image metrics at five different temperatures for ChCnf 1 (**a**), ChCnf 2 (**b**), and ChCnf 3 (**c**). Color bars represent the intensity of the images in the arbitrary units. White lines indicate the shape and position of the tumor. The unit of the axis labeling is m.

The exemplary images of this experiment reconstructed via DAS are presented in Figure 15. As we can see, the image amplitudes are decreasing with increasing temperature as expected and show almost linear behavior between temperature and image intensity in the 3D tumor region for all channel configurations.

**Figure 16:**
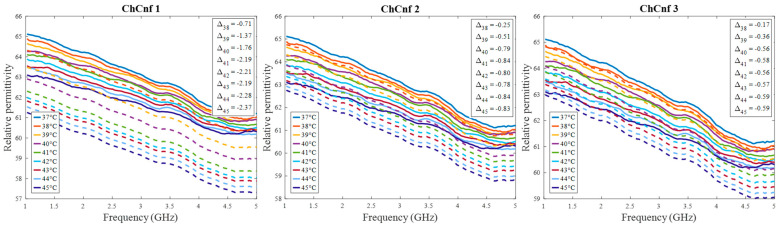
Via DAS estimated mean relative permittivity in the 3D tumor region (dashed lines) and measured values (solid lines) as a function of frequency. Differences between them at each stage at frequency 2.5 GHz are shown as $\Delta_{\vartheta }$.

Further, we estimate the relative permittivity over the frequency range 1–5 GHz (Figure 16). The estimated curves show quite smaller values of permittivity in comparison to the true curves. Analyzing Figure 16, the following observations can be made. Estimations of permittivity are much closer to the real values based on the image reconstructions via ChCnf 2 and ChCnf 3. This is due to the significantly higher amount of sensing antennas located close to the tumor (Figure 4). Noticeably, the estimation in the lower frequency range (1.0–2.5 GHz) is more accurate in contrast to higher frequencies (especially around 4–5 GHz), where significant underestimation of permittivity can be seen. This can be explained by the mathematics behind Equation (9), essentially realizing a weighted average of ${\underline{\varepsilon }}_{ref}(\omega )={\underline{\varepsilon }}_{x}(\omega ,\vartheta =37\;{}^{\circ}C)$ and ${\underline{\varepsilon }}_{bg}(\omega )$. Therefore, the slightly decreasing dispersion (decreasing temperature coefficient with increasing frequency) of the tumor mimicking material is not included in the model of Equation (9).

**Figure 17:**
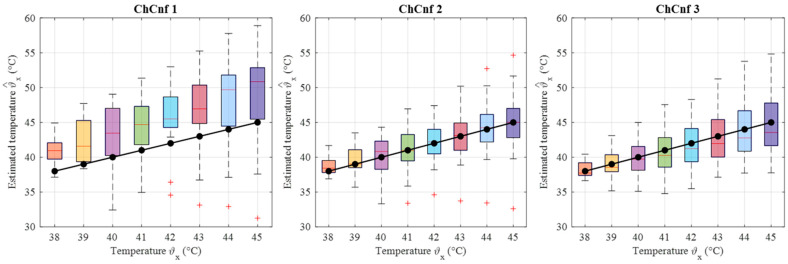
Via DAS estimated temperature of all measurement sessions. Frequency of estimation is 2.5 GHz. Black lines with markers indicate real temperature. Red lines indicate the median, red plus signs show the outliers.

Finally, the estimated dielectric properties can be converted to the estimated temperature of the tumor and compared to the real temperatures measured by the sensor. Data from 13 cooling measurement sessions are processed. The results are presented as boxplots in Figure 17. This overview confirms the observation that the lack of sensing antennas near the tumor region in ChCnf 1 is negatively influencing the accuracy of temperature estimation: more than 75% of the experimental data are overestimated in ChCnf 1. In ChCnf 2, the interquartile range is varying from the true value between ±2.3 °C and in ChCnf 3 between −3.3 and +2.8 °C. The median of the estimated temperatures corresponds really well to the real ones, especially in ChCnf 2. Clear increase in the estimated data spreading can be observed again with increasing monitoring time.

**Figure 18:**
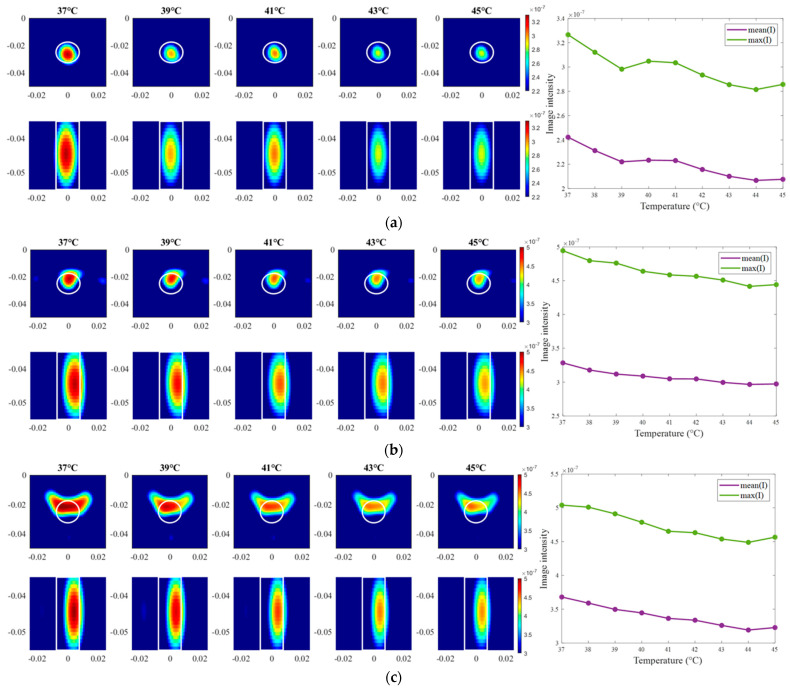
TSVD-based images in xy-slice, z = −4.5 cm (upper row), in xz-slice, y = −2.5 cm (bottom row) and corresponding image metrics at five different temperatures for ChCnf 1 (**a**), ChCnf 2 (**b**), and ChCnf 3 (**c**). Color bars represent the intensity of the images in the arbitrary units. White lines indicate the shape and position of the tumor. The unit of the axis labeling is m.

Moving forward to the measurement results processed via TSVD, images of the same exemplary cooling scenario are presented in Figure 18. It is clearly visible that the correlation between image intensity and temperature in the case of ChCnf 1 is not monotonous as expected. Small deviations can also be seen in ChCnf 2 and ChCnf 3 at 45 °C.

**Figure 19:**
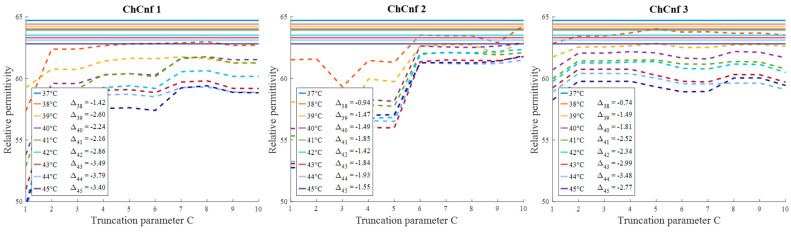
Via TSVD estimated mean relative permittivity at 1.5 GHz in the 3D tumor region (dashed lines) and measured values (solid lines) as a function of truncation parameter. Differences between them at each stage for *C* = 8 are shown as $\Delta_{\vartheta }$.

Evaluating the influence of the truncation parameter *C* on the image reconstruction, Figure 19 shows the dependence of the estimated permittivity in the 3D tumor region and the number of applied singular values. As can be seen, in ChCnf 1 and ChCnf 2, the estimated curves fall too far from the true curves using less than 7 singular values. Since Figure 19 suggests the optimal truncation belongs to the interval of 7–9 singular values, we continue to use truncation parameter *C* = 8 for further analysis.

**Figure 20:**
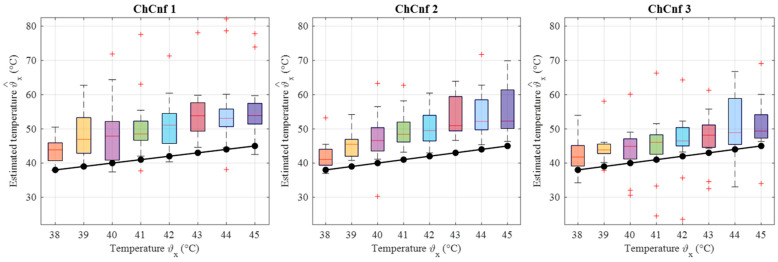
Via TSVD estimated temperature of all measurement sessions. Frequency of estimation is 1.5 GHz. Black lines with markers indicate real temperature. Red lines indicate the median, red plus signs show the outliers.

To examine the overall performance of inverse IA, the corresponding temperature estimations of all measurement sessions are calculated and presented in Figure 20. The accuracy of the estimation is significantly lower than based on DAS. All three channel configurations indicate clear overestimation of temperatures along the whole measurement procedure, while the deviations based on ChCnf 1 and ChCnf 2 are the highest. The median of the estimated values in ChCnf 3 is approximately 3.7 to 5.1 degrees Celsius higher than the true value.

## 4. Discussion

In this paper, we presented the realistic measurement setup and our methodology for non-invasive temperature estimation via MWI. The approach was experimentally validated by two types of measurements.

Firstly, the change in the dielectric properties of the tumor imitate was investigated, showing qualitative as well as quantitative correspondence to the measured values. More specifically, the mean and maximum intensities of the images in the 3D tumor region decreased with decreasing contrast between relative permittivity of background medium and tumor. Such trend was observed for both imaging algorithms applied in the data processing of this paper (Figure 9 and Figure 12). However, it can be noticed that imaging via DAS had higher accuracy in tumor localization than TSVD. This can be explained by the use of the envelope in Equation (5) as well as the complexity and sensitivity of TSVD to different interdependent parameters, e.g., sometimes outliers in TSVD results can be avoided by adjusting the truncation. The interquartile range of permittivity estimation error is less or equal to ±0.7 based on DAS and lies mainly within the range of −2 … 1 for TSVD.

In the second step of the experimental validation, the real temperature change in tumor imitate was estimated during its cooling from 45 °C to 37 °C. This temperature range accurately represents clinically approved values for HT therapy, which obviously leads to very small changes in the dielectric properties, underlying the challenge of this measurement task. Nevertheless, as can be seen from Figure 15 and Figure 18, image intensity in the tumor area was decreasing with the increasing temperature in an almost monotonous way. Detailed analysis of the estimated temperatures of all recorded sessions showed an increase in their dispersion with increasing monitoring time (Figure 17). With some exceptions, temperature estimation based on DAS imaging showed quite good results—the interquartile range of estimated values overlaps with real temperature values during the whole procedure. In results acquired via TSVD, an overestimation of temperature can be seen as well as a larger spread of the estimated values. Even though, not all sessions provided accurate temperature estimations, the overall result is quite promising, especially for the data acquired via beamforming.

One probable reason for errors in temperature estimation in the second experiment can be that permittivity is estimated in the 3D tumor region and its mean value is used for further analysis and comparison to real values. However, real temperature values inside the target tube are based only on point-like data provided by the sensors (without exact position specification), therefore excluding the distribution of the temperature. Thus, some mismatch between measured and estimated data can be present. Another option is to monitor temperature based only on the maximum value in the tumor region. Generally, reaching the upper temperature threshold for HT application (45 °C) should signal the clinician to pause or stop the heating. However, in this case, the estimation will be based only on a single voxel/pixel of the image, which makes the outcome highly dependent on the imaging algorithm. Additionally, several points were not considered in this paper, e.g., for practical reasons measurements were conducted during cooling rather than heating and dielectric properties of the healthy tissue were assumed to be temperature independent.

Next, it is worth noting that Equation (9) assumes frequency independence of the relationship; therefore, frequency-related deviations between estimated and measured relative permittivity values can be seen (Figure 10 and Figure 16). However, considering that the HT application causes really small changes in dielectric properties, these deviations remain small, as well. Additionally, since the goal of non-invasive temperature monitoring is to estimate the temperature robustly, it is sufficient to estimate the dielectric properties within an optimal frequency band. In the case of the experiments presented in this paper, the range between 2.5 GHz and 4.5 GHz was used for DAS-based estimations and 1.5 GHz for TSVD-based estimations. This choice of frequency range for temperature estimation is also in correspondence with our study [36], where the strongest temperature-induced changes in the relative permittivity were detected between 1.5 and 4.5 GHz.

Another aspect considered in this paper, is the applicability of different antenna arrangements designed for the hybrid microwave system. Comparing the investigated measurement scenarios in this paper, ChCnf 1 seems to be more disadvantageous, while the outcome of ChCnf 2 and ChCnf 3 is mostly comparable. As stated earlier, the reason for this is the lack of sensing antennas near the tumor region in ChCnf 1, which notably reduces the quality of the reconstructed images and accordingly permittivity and temperature estimation.

In comparison to our previously published work [26,28], this paper presents a realistic measurement setup for tumor temperature estimation based on UWB measurements. The most significant difference between this paper and our past publications is the reduction in the available measurements for imaging (from 3072 to just 240), since we brought our setup closer to reality and only measured stationary, i.e., without antenna or phantom rotation. Moreover, real temperature change was measured and quantitatively estimated. The results of this paper are in accordance with our earlier findings and are comparable to the work of other scientific groups in this field.

In [23], a feasibility experiment, where, similar to our first measurement, saline solution in different concentrations was used to model the variation of the tumor dielectric properties is presented. It was shown, that the location of the tumor was determined well, however, the estimated temperature values differ from the true value (∆ ≈ −6.5 °C). Such underestimation of the temperature was explained by modeling errors due to differences between 2D and 3D field computations.

One more example of an investigation towards quantitative temperature estimation is [24], where saline solution was heated to different temperatures. This study revealed that the trend of reconstructed dielectric parameters correlated well with the measured ones. But the quantitative difference between estimated and measured permittivity values showed overestimation of permittivity by 2 … 4 in the HT temperature range. Two reasons of error were identified: heat conduction from the relatively hot tumor mimicking material into the phantom, causing deviation between measurement setup and numerical model, and regularization of TSVD.

In future works, the estimation approach has to be adapted to differential imaging, thermal effects have to be investigated in more detail and considered in the estimation procedure, and the regularization parameters of the inverse scattering should be adjusted. Finally, a complex heterogeneous phantom structure mimicking different neck organs introduced in [30] is planned to be used in the experiments.

## Data Availability

The datasets presented in this article are not readily available because the data are part of ongoing PhD study. Requests to access the datasets should be directed to the authors.
